# A case of disseminated cutaneous Kaposi sarcoma in an immunocompetent patient

**DOI:** 10.1186/1471-2334-14-S7-P1

**Published:** 2014-10-15

**Authors:** Maria Isabela Sarbu, Mircea Tampa, Ilinca Nicolae, Clara Matei, Teodor Poteca, Vasile Benea, Simona Roxana Georgescu

**Affiliations:** 1 Clinical Hospital of Infectious and Tropical Diseases “Dr. Victor Babeş”, Bucharest, Romania; 2 Carol Davila University of Medicine and Pharmacy, Bucharest, Romania; 3 Colentina Clinical Hospital, Bucharest, Romania

## Background

Kaposi's sarcoma (KS) is a tumor derived from the endothelial cell lineage caused by Kaposi sarcoma-associated virus (KSHV), also known as human herpes virus 8 (HHV-8). Four subtypes of KS have been described: classical KS, African endemic KS, immunosuppression-associated KS and AIDS-associated KS. Over 95% of the lesions have been found to be infected with HHV-8, regardless of the clinical subtype. Classical KS usually occurs in elderly men from the Mediterranean region. It is a chronic, slowly progressing disorder, usually confined to the skin, which only rarely affects other organs.

## Case report

We report the case of a 56-year-old male patient who addressed to our clinic presenting extensive violaceous plaques comprising both feet and lower half of the calves, the right hand, the dorsum of the left hand, as well as smaller lesions located on the lower and upper extremities and the torso. Several violaceous firm nodules of various diameters were scattered on the surface of the plaques. The lesions were associated with extensive oedema and therefore the functionality of the right hand was severely impaired and the patient had walking difficulties. The patient asserts that the lesions had first occurred two years prior to the presentation on the lower extremities and rapidly enlarged. He was diagnosed with lichen planus and was treated with topical and systemic glucocorticoids, which helped reduce the oedema but had little effect on the cutaneous lesions. When the systemic glucocorticoid treatment was ceased, the oedema rapidly recurred.

The HIV testing turned out negative. Laboratory findings were within normal range. A biopsy was taken from one of the lesions and the clinical suspicion of Kaposi's sarcoma was confirmed. The patient was treated with radiotherapy and the oedema rapidly reduced, the patient regaining the functionality of his hands and feet.

## Conclusion

The classical form of Kaposi's sarcoma is usually a chronic, indolent disorder, slowly progressing over a period of several years or decades. The particularity of the case is the rapid progression in an otherwise healthy patient.

## Consent

Written informed consent was obtained from the patient for publication of this Case report and any accompanying images. A copy of the written consent is available for review by the Editor of this journal.

